# Altered histone modifications in *Aedes aegypti* midguts following Rift Valley fever virus exposure

**DOI:** 10.1038/s41598-026-37729-y

**Published:** 2026-01-29

**Authors:** Hunter A. Ogg, Zoey M. Mikol, David C. King, Chad E. Mire, Zeyad Arhouma, Erin Osborne Nishimura, Rebekah C. Kading, Corey L. Campbell

**Affiliations:** 1 Center for Vector-borne Infectious Diseases, Fort Collins, USA; 2 Department of Microbiology, Immunology, and Pathology, Colorado State University, Fort Collins, CO 80523 USA; 3 Department of Biochemistry and Molecular Biology, Colorado State University, Fort Collins, CO 80523 USA; 4 United States Department of Agriculture, Agricultural Research Services, National Bio and Agro-defense Facility, Foreign Arthropod-Borne Animal Diseases Research Unit, Manhattan, KS USA

**Keywords:** Mosquito, Vector competence, *Culicidae*, Chromatin immunoprecipitation sequencing, Differential expression, RNA-Seq, ChIP-Seq, Histone modifications, Genetics, Microbiology, Molecular biology

## Abstract

When arthropod-borne viruses (arboviruses) are delivered to vector mosquitoes in infectious bloodmeals, viral components interact with host proteins to hijack cells and initiate replication. The extent to which arbovirus infection alters mosquito host transcriptional regulatory processes is currently unknown. We hypothesized that histone modifications would be altered in mosquitoes exposed to Rift Valley fever virus (RVFV MP12). H3K27ac and H3K9me3 marks were interrogated using CUT&RUN in a mosquito species that has a predicted dissemination barrier, *Aedes aegypti*. Global H3K27ac peaks showed progressive depletion over time compared to bloodfed controls. Gene set enrichment analysis revealed that immune response transcripts were enriched at 1 and 3 days post-feeding (dpf). For virus-exposed samples, the highest proportion of DEGs proximal to histone marks occurred with depletion of repressive H3K9me3 peaks at 3 dpf. Associated DEGs included transcription factors, secondary messengers and processes affecting cell polarization. Analysis of midguts after a non-infectious bloodmeal versus sugar-fed controls revealed global changes to H3K27ac and H3K9me3 marks, as well. Differential H3K27ac marks were proximal to one quarter of all DEGs at 1 dpf, consistent with an important role of H3K27ac in bloodmeal digestion. Together, these results demonstrate that H3K27ac and H3K9me3 patterns are altered upon virus exposure in a complex interplay that could be due to viral manipulation or host defense.

**Supplementary Information:**

The online version contains supplementary material available at 10.1038/s41598-026-37729-y.

## Introduction

Vector-borne disease outbreaks, particularly those caused by arthropod-borne viruses (arboviruses), have increased in frequency and intensity in recent years^1^. Mosquito-borne viruses are unique in that they must successfully replicate in an alternating fashion among invertebrate and vertebrate hosts. As intracellular pathogens, they interact with host proteins to modulate cellular processes and push metabolic activity in favor of replication and viral assembly. Regardless of whether the host is a mammal or mosquito, selective repression of transcriptional activity occurs^2–4^, which is a hallmark of cellular hijacking to enable efficient viral propagation. In vector mosquitoes, immune tolerance and resistance allow viral replication without pathological signs. Virus replication is affected by and, in turn, influences host genomic regulation (reviewed in^5^. Though histone modifications have been best studied with DNA viruses, recent evidence has also implicated histone modifications in the efficacy of RNA virus infection, including flavivirus infection of mosquitoes^6–9^. These alterations could underpin features of host tolerance and resistance in vectors.

Histone modifications occur during development^10^ across taxa and are also important during the innate immune response^11,12^. Histone 3 lysine 27 acetylation (H3K27ac) is a mark of accessible chromatin, which facilitates entry and binding of proteins to initiate transcriptional activation. H3K27ac can also serve as a marker for enhancer activity and be perturbed upon pathogen infection^13,14^. In particular, H3K27ac levels are elevated during Zika virus infection in *Ae. aegypti* cell culture via acetyl transferase CBP activity^6^. In contrast, histone 3 lysine 9 trimethylation (H3K9me3) is associated with heterochromatin or silenced genes (reviewed in^15^; though, during virus infection, specific genes may be derepressed^16^. Though the details of the overall structural features of mosquito chromatin are largely unknown^17^, recent efforts have begun to explore distinct elements, such as the importance of histone modifications^18,19^. Other studies used computational approaches to identify *cis* regulatory elements (CREs) that differed between dengue virus susceptible and resistant *Ae. aegypti* strains^20^. Still, much remains to be understood of the role of CREs and chromatin modifications in the vector response to arbovirus infection.

Here, we focus on Rift Valley fever virus (RVFV, *Phlebovirus riftense*, family *Phleboviridae*), using the MP12 vaccine strain as a model to explore the link between genome regulatory marks and gene expression changes. RVFV is a zoonotic pathogen of concern in sub-Saharan Africa^21,22^. Our previous studies established *Ae. aegypti* as a suitable model for study of RVFV-mosquito interactions using the MP12 vaccine strain^4,23–25^. Nevertheless, it is less susceptible than *Culex tarsalis*, due to a predicted dissemination barrier^23,25,26^. Previous studies provided support for the idea that *Ae. aegypti* mounts an effective innate immune response against RVFV compared to the much more competent vector, *Culex tarsalis*, as evidenced by viral infection kinetics and differential expression of a key signaling gene, *dishevelled*^4,23^. Though *Ae. aegypti* is well known as a competent vector for a variety of arboviruses, in the current project we sought to define genomic features of anti-viral defense that underpin dissemination barriers to infection.

**Fig. 1:**
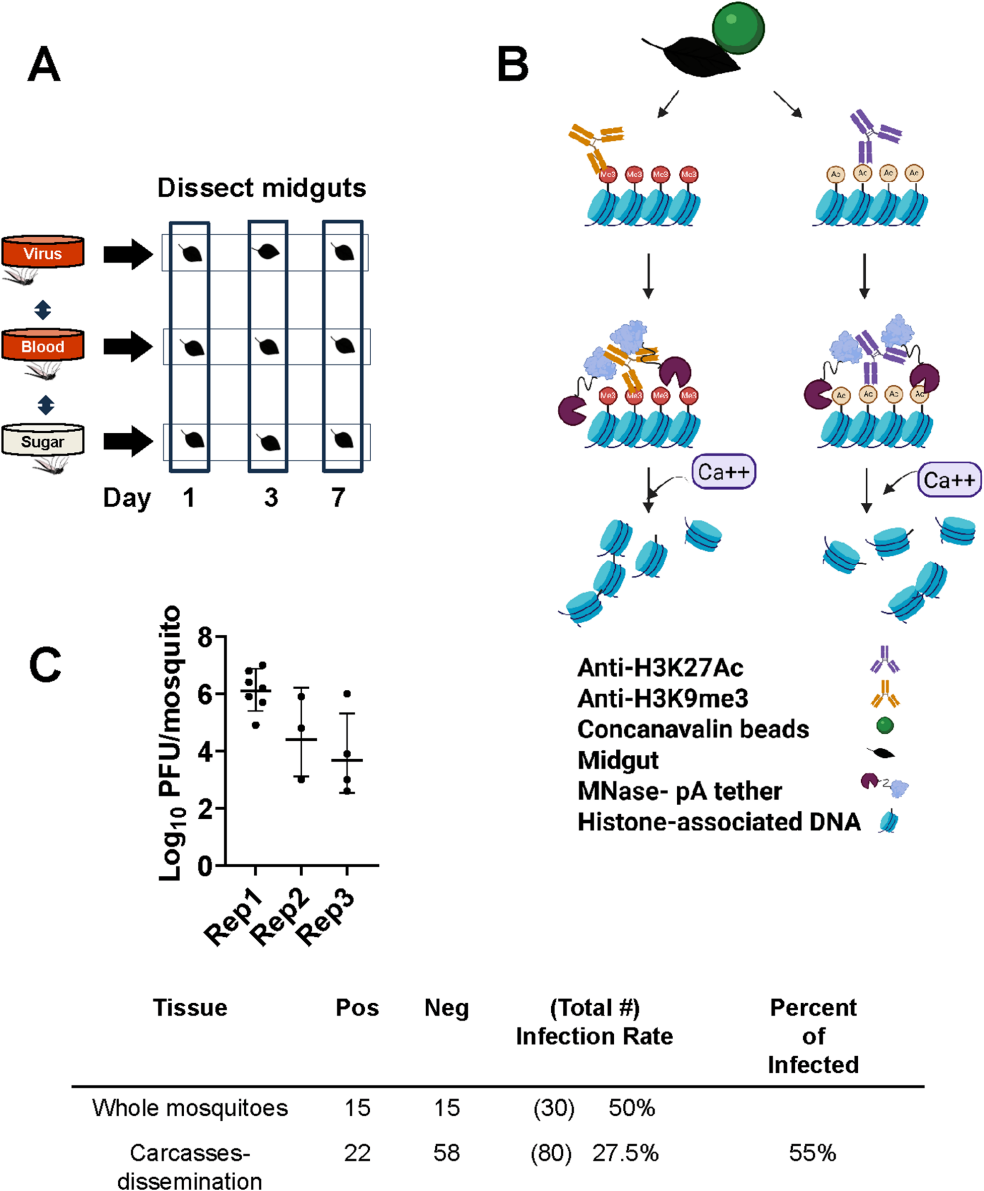
CUT&RUN enables capture of histone modifications. (**A**) Two major experiments were done, RVFV MP12 vs. bloodfed (RVFV v BF) or bloodfed vs. sugar-fed (BF v SF) at 1, 3, or 7 dpf. Pools of 20 midguts were used for each sample in triplicate biological replicates of CUT&RUN or RNA-Seq. CUT&RUN libraries with inserts < 55 nts were removed from analysis. DNA Peaks from merged libraries were called against input controls. (**B**) For CUT&RUN, fixed midguts were bound to magnetic beads coated with concanavalin A (green), permeabilized with digitonin, and treated with antibodies specific for H3K27Ac or H3K9me3. Protein A/G- micrococcal nuclease fusion protein cleaved DNA at antibody binding sites for release into the supernatant, then subjected to purification and library preparation. Created in BioRender. Lab, K. (2025) https://BioRender.com/9y5ab72 (**C**) RVFV titers from virus-exposed mosquitoes collected at 7 dpf provide a measure of infectious viral loads. Graph indicates range of titers for whole mosquitoes (with intact midguts) across 3 experimental replicates. Top row- Infection rates of representative whole mosquitoes shown in graph above. Bottom row- Infection rates determined from representative carcasses following removal of midguts for CUT&RUN indicate dissemination rates.

To explore the relationship between gene expression changes and genomic regulatory regions, we chose to characterize H3K27ac and H3K9me3 histone modifications in midguts of RVFV-MP12-exposed *Ae. aegypti* adult females and compare them to signatures of gene expression(Fig. 1), using antibodies validated in mosquitoes^19^. Midguts are often the first site of arbovirus replication. Analysis of chromatin marks in mosquito tissues is made possible by recent advances in chromatin immunoprecipitation sequencing, specifically Cleavage Under Targets and Release Using Nuclease (CUT&RUN). This technique and similar approaches require substantially less biological material and have improved signal-to-noise, compared to traditional chromatin immunoprecipitation methods^27–29^(Fig. 1B). Midgut gene expression changes that occur following exposure to RVFV MP12 were paired with companion analyses of mosquitoes that had received a bloodmeal alone compared to sugar-fed controls. We followed up these studies with interrogation of H3K27ac and H3K9me3 signatures. Our initial hypothesis was bloodfeeding induces gene expression and associated chromatin modifications that set the metabolic foundation for successful arbovirus infection^30^. Moreover, we also predicted histone modifications would be altered following exposure to RVFV MP12, as evidenced by association with differentially expressed genes. We expected that important regulatory changes may occur prior to detection of infectious virus, therefore we chose to assess midguts at early timepoints post-infection (1, 3 and 7 days) in virus-exposed midgut pools. We focus first on changes that occurred following RVFV exposure and follow up with control experiments. Our results revealed that differentially expressed genes (DEGs) were proximal to altered H3K27ac or H3K9me3 marks at 3 dpf. Global H3K27ac signatures were substantially altered by 7 dpf but were tied to only a few DEGs.

## Results

### Gene expression upon RVFV exposure

**Fig. 2:**
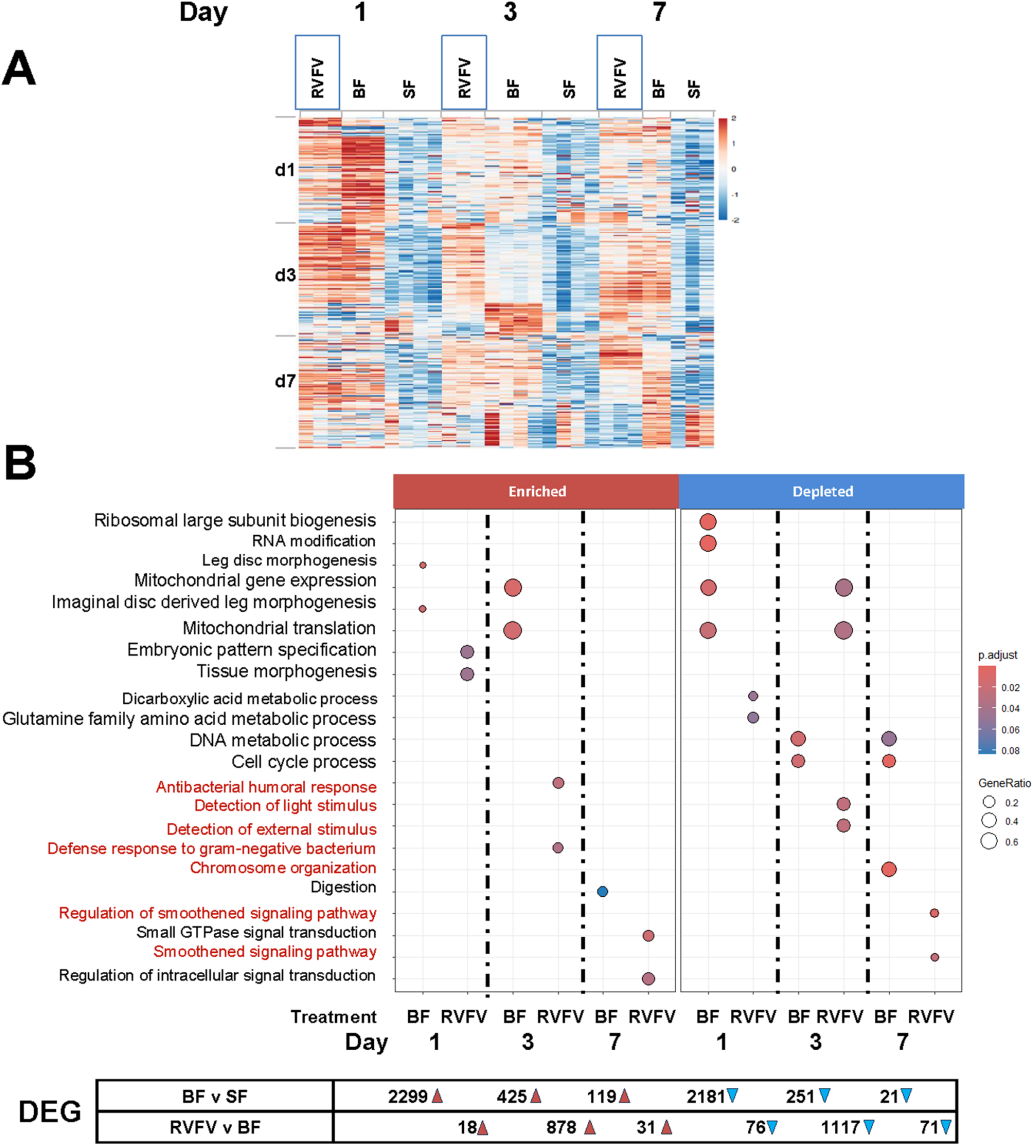
Differential gene expression profiles at 1, 3 and 7 days post-treatment. (**A**) Heatmap of bulk RNA-Seq data pools of 20 midguts per timepoint were ranked by the top 100 genes of the RVFV treatment group on the indicated dpf. Columns group individual biological replicate z-scores of RPKM values for RVFV vs. BF by treatment group (RVFV, BF, SF), then by timepoint. Number of replicates ranged from 2 to 4. (**B**) GO-GSEA dotplot of RNA-Seq differential expression categories for 1, 3 and 7 days post-treatment BF (bloodfed vs. sugar-fed) and RVFV (RVFV-exposed vs. BF). GeneRatio refers to the proportion of the gene set of interest divided by the total number of genes. Highlighted biological processes are noted in red font. Numbers below indicate DEGs that were enriched (red triangle) or depleted (blue triangle).

To gain insight into transcriptional profiles following RVFV-MP12 exposure, *Ae. aegypti* were provided an infectious oral bloodmeal, and pooled midguts were processed for RNA-Seq at 1, 3, and 7 days post-feeding (Fig. 1A, Supplementary Table S1, Supplementary Fig. S1). Viral bloodmeal titers were 6.9–7.8 log10 plaque-forming units (PFU) per ml. Virological confirmation showed that 50% of whole mosquitoes carried infectious virus at 7 days post-feeding (dpf), and a representative set of carcasses showed that about 55% of infected mosquitoes had disseminated infections (Fig. 1C). This is consistent with previous studies, wherein wildtype viral antigen, which does not necessarily represent infectious virus, disseminated in *Ae. aegypti* by 6 dpf^23,25^. Also, this is later than what occurs in the more competent vector, *Culex tarsalis*^31^. For the RVFV v bloodfed (BF) comparison at 1 dpf, virus-exposed midgut pools had 94 DEGs(FDR < 0.10, Fig. 2, Supplementary Table S2) compared to blood-fed controls. Gene set enrichment analysis (GSEA) using our custom annotation was done to calculate the normalized enrichment score (NES) for each functional category across all the collection times (Supplementary Table S3)^32^. NES analysis indicated that overall signaling and immune response genes were enriched at 1 dpf, as was the broad category of replication, (DNA) repair, transcription, translation^33^. As the infection progressed, by 3 dpf, there were 1995 total DEGs. This functional category continued to be enriched, along with cytoskeletal and structural, immune and proteolytic pathways (Fig. 2B, Supplementary Fig. S2, Supplementary Table S3). By 7 dpf, 102 DEGs were differentially expressed in the RVFV-exposed group, with 71 DEGs depleted, and just 31 transcripts enriched over BF controls (Fig. 2, Supplementary Table S2). Custom GSEA analysis indicated that replication, (DNA) repair, transcription, translation transcript categories were still over-represented in virus-exposed midguts (Supplementary Table S3), which would be consistent with continued viral replication at this timepoint. However, immune response, proteolysis and metabolic functional categories were significantly under-represented, consistent with select transcriptional repression expected during virus-associated modulation of gene expression^34,35^.

GSEA analyses are often limited by the number of genes in a transcriptome that are assigned functional terms. Because our custom annotation is limited in terms of scope and functional category, we applied gene ontology (GO) term GSEA (GO-GSEA) analysis based on eggNOG mapper orthological assignments^36^. GO-GSEA provided corroborative evidence that anti-microbial and signaling responses were enriched at 1 and 3 days following RVFV exposure (Fig. 2B, Supplementary Fig. S2). By 7 dpf, GTPase signaling and overall regulation of signaling processes were significantly enriched in RVFV exposed midguts compared to BF controls at 7dpf (Fig. 2B, Supplementary Table S2). Lastly, constituents of the smoothened signaling pathway were depleted at 7 dpf but unaffected upon bloodfeeding alone. Smoothened is a signaling pathway associated with the Hedgehog/Gli signaling pathway, which is responsible for maintenance of cell polarity and tissue development^37^. To further support the role of the smoothened signaling pathway, the *Drosophila melanogaster* Ci/Gli consensus (GACCACCCA) was searched against promoters of the *Ae. aegypti* genome. Overrepresentation analysis of 3221 unique genes across the genome with Ci/Gli promoters indicated that negative regulation of chromatin and epigenetic regulation as well as negative regulation of DNA-templated transcription were major processes controlled by this promoter(Supplementary Fig. S3).

### Gene expression upon a noninfectious bloodmeal

To differentiate the effects of virus exposure from those of bloodfeeding alone, additional experiments were performed to reveal effects on differential gene expression and histone modifications. By providing proteinaceous nutrients critical for egg-laying, bloodfeeding is crucial for reproduction. Because arbovirus infection of mosquitoes intrinsically requires a bloodfeeding event, and it is therefore intricately tied to vector competence, we hypothesized that bloodfeeding alone modulates midgut gene expression to favor mosquito permissiveness to virus infection^30^. Comparison of blood-fed vs. sugar-fed (BF v SF) mosquito midguts at 1 day post-feeding (dpf) revealed 4480 DEGs (FDR, < 0.10, Fig. 2, Supplementary Fig. S1 and S4, Supplementary Table S2). The expression of digestive enzymes is well documented^38^, cellular processes with the lowest adjusted p values (padj) at each timepoint are highlighted here. Multiple RNA processing and cellular biogenesis pathways were modulated at 1 dpf. By 3 dpf, the bloodmeal was completely digested; nevertheless mitochondrial processes remained elevated (Fig. 2B). By 7 dpf, processes affecting chromosome organization and cell cycle progression were modulated compared to sugar-fed controls (Supplementary Fig. S4).

### Altered H3K27ac profiles in RVFV v BF

**Fig. 3:**
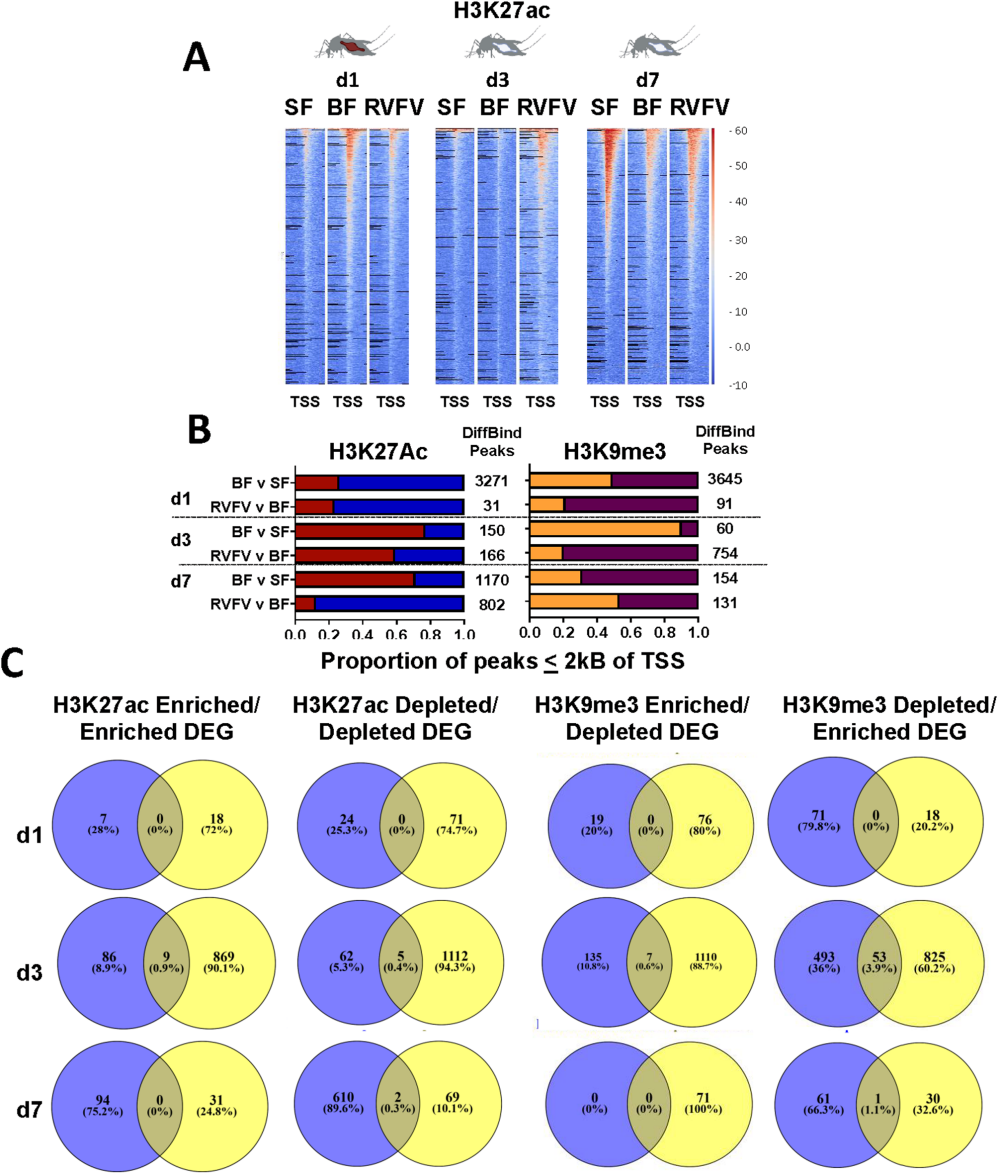
Midgut histone modification profiles. (**A**) Representative H3K27ac peak heatmaps of aligned reads at 1, 3 and 7 days post-treatment show global trends within 2 kB of TSS (x axis). Reads were standardized to RPGC. Y-axis indicates RPGC of input-subtracted read alignments. Created in BioRender. Lab, K. (2025) https://BioRender.com/7idyfsl (**B**) Proportion of DiffBind peaks within 2 kB of TSS (FDR < = 0.10) for H3K27ac, (red = enriched peaks compared to control, blue = depleted peaks compared to control) and H3K9me3 (orange = enriched, maroon = depleted). Total numbers of DiffBind peaks within 2 kB of TSS listed to the right of the bars. (**C**) Venn diagrams show intersection of select differential histone peak groupings (purple) adjacent to unique DEGs (yellow). H3K9me3 marks have the highest number of coordinated DEGs.

Next, to determine whether gene expression changes associated with bloodfeeding and RVFV exposure were tied to H3K27ac and H3K9me3 modifications, we performed CUT&RUN on midguts following a bloodmeal and at 1, 3, 7 dpf following exposure to RVFV MP12 (Supplementary Tables S4 and S5). RVFV exposure led to incremental changes to global H3K27ac patterns over time that were lower than that of BF at 1 dpf and gradually increased over the course of infection (Fig. 3A, Supplementary Fig. S5). By 7 dpf, RVFV-exposed midguts showed slightly higher levels of global acetylation patterns than BF controls (Fig. 3A). Upon analysis of statistically significant differences, a striking pattern emerged. There were 31 significantly different H3K27ac peaks proximal to transcription start sites (TSS) at 1 dpf (Fig. 3B). Of these, 24/31 (77%) peaks were depleted in RVFV-exposed and just 6 peaks showed higher levels than BF controls (Fig. 3C). Though the number of altered H3K27ac peaks were much greater following a bloodmeal alone, the overall proportion of depleted/enriched peaks were similar (74% peaks depleted).

By 3 dpf, an overall increase in H3K27ac enrichment occurred for both treatment groups (red bars, Fig. 3B). Nevertheless, the proportion of significantly enriched H3K27ac peaks was reduced in RVFV-exposed (41%) compared to BF v SF treatment group(23% depleted, Fig. 3B). This trend was even more dramatic at 7 dpf, wherein acetylation patterns in RVFV-exposed mosquitoes showed a marked reduction compared to BF controls. In particular, 705/802 (88%) significant peaks were depleted in RVFV exposed midguts compared to just 29% in the BF v SF group (Fisher’s exact test, *p* < 0.0001, Fig. 3B, Supplementary Table S5). In RVFV-treated samples, genes of interest proximal to TSS showed changes to H3K27ac occupancy over time; these genes were expected to affect cell morphogenesis at 1 dpf, regulation of transcriptional processes at 3 dpf and membrane transport by 7 dpf (Supplementary Fig. S6). We currently cannot distinguish whether these effects were due to virus-associated modulation or the host response.

Bloodfeeding alone was associated with changes to H3K27ac peak patterns that varied over the timecourse. Global distributions of H3K27ac patterns increased upon bloodfeeding compared to SF but waned by 3 dpf (Fig. 3A). Of 3271 quantitatively differential TSS-proximal peaks at 1 dpf(Supplementary Table S4, Fig. 3B), just 24% were enriched in BF compared to SF controls. At 3 dpf, the overall numbers of differential peaks were lower, however the proportion of those that were enriched increased to 77%. By 7 dpf, the sugar-fed group shows markedly higher levels of acetylation than either treatment group, which may be an indicator of aging in the absence of a bloodmeal (Fig. 3A). GO-GSEA of genes proximal to TSS showed categories that were consistent with those of RNA-Seq data (Supplementary Fig. S4), with genes affecting RNA metabolism showing modulation of H3K27ac marks at 1 dpf. These altered genomic regions supported changes to regulation of metabolic processes by 3 dpf. Finally, by 7 dpf, genes responsible for controlling chromosomal reorganization were identified proximal to H3K27ac sites.

Gene set over-representation analysis (GSORA) for genes proximal to H3K27ac marks (MACS2 peaks) showed changes over time in blood-fed vs. sugarfed controls(Supplementary Fig. S7). In particular, at 1 dpf, peaks proximal to genes associated with chromosomal organization and histone modifications were altered in the BF treatment group, a pattern that shifted to catabolic and amide biosynthetic processes by 3 dpf (Supplementary Fig. S7). These associations continued over time. In contrast, at 7 dpf, both SF and BF samples showed H3K27ac marks proximal to genes involved in several intracellular transport categories, including vesicle-mediated, amide and peptide transport (Supplementary Fig. S7).

### RVFV-associated DEGs with altered histone peak patterns

Association of DEGs with H3K27ac marks varied over the time course. In RVFV-exposed midguts at 1 dpf, no differentially expressed genes had changes to proximal H3K27ac marks. However, at 3 dpf, 14 of 2023 DEGs supported regulation with enriched H3K27ac marks and enriched DEGs, as well as the converse set(Fig. 3C). These coded for several genes with predicted transcriptional regulatory or signaling function(Supplementary Table S7). By 7 dpf, there were 7/102 DEGs with depleted promoter-proximal acetylation patterns (Supplementary Table S7), 5 of which had enriched RNA levels, consistent with active gene expression. Of these 5 genes, 2 code for proteins that facilitate protein-protein interactions on cellular membranes, including AAEL005849 (synaptic vesicle protein) and AAEL019495 (pleckstrin/PDZ domain). Predicted gene function, coupled with transcript enrichment support the idea that these genes are proviral in supporting virus assembly or intracellular transport. A third gene, AAEL021746 (LysM-TLDc domain), which is predicted to regulate oxidative stress, showed enrichment of transcripts and depletion of H3K27ac marks^39^. The 2 genes with transcript depletion coupled with depletion of H3K27ac marks in RVFV-exposed midguts are predicted to have anti-viral function. One gene was a cytochrome P450 (CYP307A1, AAEL009762) and the second was a leucine-rich repeat (AAEL023746), which could be a novel pattern-recognition receptor. To validate expression of these transcripts, relative RT-qPCR was performed on 5 of 7 DEGs at 7 dpf. Four of those 5 showed the expected directionality of differential expression (Supplementary Table S7).

Altered H3K9me3 peaks at 3 dpf were associated with the most DEGs (Fig. 3C, Supplementary Table S7). H3K9me3 marks may be associated with poised enhancers or transcriptional repression, depending on adjacent chromatin signatures^40,41^. Analysis of differentially bound H3K9me3 peaks by DiffBind showed depletion of peaks following RVFV exposure at 1 and 3 dpf (Fig. 3B, maroon bar, Supplementary Table S6, Supplementary Fig. S9), which was consistent with transcriptional de-repression. No DEGs overlapped with H3K9me3 peaks at 1 dpf. By 3 dpf, however, 663 unique DEGs showed peaks within 2 kB of TSS (Supplementary Table S6). Specifically, of 754 differentially bound peaks in the 3 dpf RVFV vs. BF group, 53 enriched DEGs showed depletion of proximal H3K9me3 peaks (Fig. 3C, Supplementary Table S7). ORA analysis of enriched DEGs proximal to depleted H3K9me3 marks revealed genes that coded for transcription factors, modulators of kinase activity consistent with secondary messenger systems and cellular cationic channel activity (Supplementary Figure S10). Therefore, instead of controlling part of viral repression of gene expression, as expected, our data was consistent with the idea that depletion of H3K9me3 peaks at 3 dpf supported derepression of gene expression. By 7 dpf, just one DEG in the RVFV-exposed group, AAEL021746, showed significant depletion of H3K9me3 peaks.

### Post-bloodmeal gene expression changes coincide with histone modifications

A key goal of this study was to identify associations between gene expression changes and histone marks. One day following a noninfectious bloodmeal, about one fourth of all DEGs (1052/4480) showed proximal H3K27ac marks (Supplementary Table S5). There were 512 depleted DEGs among those that had altered peaks. Of those, 383/512 showed corroborative depletion of H3K27ac marks. However, for the enriched peaks, just 84/540 enriched DEGs showed the expected pattern of peak enrichment. Supplementary Fig. S8 shows the pattern of DEGs relative to promoter-proximal DiffBind peaks across all 3 chromosomes. This suggests that gene expression differences cannot be explained by H3K27ac enrichment alone. At later timepoints following a bloodmeal alone, fewer enriched DEGs showed corroborative H3K27ac enrichment, 6/676 and 11/140 at 3 and 7 dpf, respectively.

### Enhancers

Enhancers are long-range regulatory elements that activate or repress gene expression. H3K27ac marks are most often associated with enhancer activation of gene expression, whereas H3K9me3 enhancers are typically associated with transcriptionally repressed regions^41–43^. To explore potential involvement of enhancers, we interrogated our datasets for the presence of H3K27ac or H3K9me3 marks that were 50,000 to 200,000 nts away from TSSs. H3K27ac enhancers were associated with bloodfeeding at 1, 3 and 7 dpf (Supplementary Fig. S11). At 1 dpf, the genes with these putative enhancer elements coded for RNA processing and metabolic changes (Supplementary Fig. S11A). In contrast, H3K27ac enhancers were enriched upon RVFV exposure only at 3 dpf.

Putative H3K9me3 enhancers were generally depleted upon bloodfeeding at 1 and 3 dpf (Supplementary Fig. S11B, maroon bar), which would be consistent with large-scale changes in metabolic needs, necessitating broad scale re-structuring of chromatin concomitant with gene expression changes. For RVFV exposed samples, enhancers were enriched at 1 and 7 dpf, which supports our hypothesis of virus-induced transcriptional repression. However, this interpretation is complicated by the lack of altered enhancer peaks at 3 dpf.

## Discussion

Global changes in H3K27ac marks occurred in a layered fashion in *Ae. aegypti* midguts upon receiving a non-infectious bloodmeal or concomitant RVFV MP12 exposure. At 1 dpf, BF v SF and RVFV v BF H3K27Ac peak profiles showed similarity in the overall proportions of depleted and enriched peaks (Fig. 3B). Blood-feeding alone led to an increase in enriched acetylation marks by 3 dpf, but proportions showed a slight reduction in RVFV-exposed midguts. By 7 dpf, RVFV exposed midguts showed significant depletion of H3K27ac marks relative to BF controls(Fig. 3B). These results were broadly corroborated in the RNA-Seq data, with 70% of DEGs being depleted in the RVFV-exposed group compared to BF controls at 7 dpf (Fig. 2B). However, overall, altered marks were contiguous with just a few statistically significant gene expression changes (Fig. 3C). Such association may have been limited by the timepoints tested or by the lack of assessment of other key chromatin modifiers or transcriptional regulators.

H3K9me3 marks are typically associated with gene silencing. Due to the expected selective reduction in gene expression upon viral infection^44^, we initially hypothesized that H3K9me3 marks would be enhanced during virus infection, consistent with repression of gene expression. Instead, significant depletion of H3K9me3 marks was observed in the 3 dpf RVFV vs. BF group, which is consistent with derepression of gene expression. Functional groups of DEGs adjacent to depleted H3K9me3 marks code for modulators of transcription factors, kinase activity and cellular polarization (Fig. S10). Without further experimentation, we are unable to determine whether changes to gene expression are proviral or defense-related.

Identification of validated transcriptomic biomarkers of arbovirus infection across *Ae. aegypti* strains has been challenging, even for a single type of virus^45^. This is likely due to the dependency of vector competence on genetic interactions between vector host and viral genotypes^46,47^, as well as environmental factors. Therefore, study of genomic regulatory mechanisms could reveal genomic regions that underpin vector competence phenotypes. Importantly, study of transcriptional regulation in vivo is critical, because histone modification patterns are not synonymous across cell culture and mosquito tissues^48^. To assess whether RVFV-associated gene expression changes were similar to those reported elsewhere, we compared our DEGs to those from other transcriptomic studies of midgut-associated arbovirus-responsive genes, while allowing for co-modulation at a different timepoint and altered directionality of expression. For studies of dengue-1 virus (DENV1), 48 DEGs from Raquin et al. were cross-listed with RVFV-responsive genes reported here^49^ (Supplementary Table S2). A study of DENV2 resistant and susceptible *Aedes* strains showed a number of genes with opposite expression patterns^45^. In our study, 2 genes from the 1 dpf timepoint were co-regulated in midguts from a DENV resistant *Ae aegypti* strain; these genes coded for a choline/ethanolamine kinase (AAEL008853) and transferrin (AAEL015458), an iron-binding protein that is induced as part of the immune response^45,50^. Lastly, 13 DEGs from Dong et al., wherein chikungunya virus-associated responses were assessed, were co-modulated with DEGs reported here^51^ (Supplementary Table S2). These data support the idea that *Ae. aegypti* may have some similarities in response across arboviruses.

Our study was designed to identify associations between histone marker changes and DEGs. Nevertheless, in the literature, it is well recognized that histone marks alone are not strong predictors of transcriptional changes^52–54^. Rather, they are one layer in a highly complex, multilayered regulatory landscape, where histone marks provide or prevent accessibility to chromatin, and other chromatin modifiers, transcription factors and non-coding RNAs work with Mediator and RNA polymerase to carry out gene expression.

Here, RVFV-associated host gene expression changes for a variety of functional categories occurred over time. For example, stimulation of cellular differentiation processes at 1 dpf were consistent with metabolic changes that might be required to support production of viral replication complexes(Fig. 2C)^55^. These were coupled with repression of transcripts coding for dicarboxylic acid and glutamine metabolic processes, which could affect cellular signaling^56^. By 3 dpf, cellular processes specific to cell polarization processes were modulated (detection of light stimulus, phototransduction), as well as transcripts that control mitochondrial gene expression/translation (Supplementary Fig. S3). By 7 dpf, signal transduction processes were repressed for those in the RVFV-exposed group but not upon blood-feeding alone. In particular, components of the smoothened signaling pathway were depleted in the RVFV-exposed group(Supplementary Table S4, Fig. 3B). Smoothened is a frizzled class G-protein coupled receptor that acts in the hedgehog/Gli (C/Gli) pathway to regulate cell polarity^37,57^. Multiple lines of evidence indicate that arboviruses preferentially replicate in polarized cell types^58–60^. RVFV enters polarized mammalian cells more efficiently through the apical surface, and viral particles mature near basolateral membranes^61^. The smoothened pathway has antiviral characteristics in mammalian neuronal cells, which are polarized, therefore repression may favor viral propagation^62^. Though genes with C/Gli promoters were present across the sample sets (Supplementary Table S8), the gene set was not detected by GO-GSEA until 7 dpf. Further characterization will have to be done to determine the role of the smoothened pathway during RVFV infection.

Midguts are comprised of mixed cell populations with differing competency for virus infection^55^. The results captured here do not distinguish between different cell types or infection status. In addition, midgut pools were used for each replicate sample, thus reducing the high level of variability expected for individual mosquitoes^4^. Therefore, the patterns represent overall trends associated with viral exposure.

We demonstrated enrichment of immune response transcripts at 1 and 3 dpf, which were above and beyond those induced by a noninfectious bloodmeal^63^. RVFV-responsive genes of interest included defensin (AAEL003832, AAEL003857), cecropin (AAEL029046, AAEL029047), as well as transcriptional regulators that control immune transcriptional responses. However, by 7 dpf, immune response transcripts, e.g., pattern recognition proteins (AAEL001414, AAEL001420, AAEL010125, AAEL023746, AAEL024406) were depleted relative to BF controls. A more competent vector might have less of a pronounced immune response at these early timepoints. *Ae. aegypti* has an intermediate level of competency for RVFV infection and transmission. Vector competence is much less than that of *Cx tarsalis* and higher than that of *Cx pipiens quinquefasciatus*^23,64^. GO-GSEA analysis showed that defense processes were enriched at 1 and 3 dpf but repressed by 7 dpf. These observations were consistent with active immune response gene expression early in infection as part of overall intrinsic immune responses, which are key determinants of mosquito vector competence^65^. We would expect that a more susceptible mosquito species would show immune response repression earlier in the course of infection. However, further experiments are needed to sort out chromatin modulation across susceptible and resistant phenotypes.

Interestingly, one DEG, AAEL021746 (LysM-TLDc domain), which was slightly enriched in RVFV exposed midguts at 7 dpf, showed significant depletion of both H3K9me3 and H3K27ac peaks. This gene also has a C/Gli promoter motif (Supplementary Table S8). LysM-TLDc containing proteins, e.g. human NCOA7 (Nuclear receptor coactivator 7), have been shown to interact with vacuolar-ATPase, which promotes endoplasmic vesicle acidification to impair infection of influenza A virus, human immunodeficiency virus and SARS-CoV^66–68^. This evidence suggests that LysM-TLDc domain containing proteins, such as AAEL021746, have anti-viral activity.

In the current study, over one quarter of all DEGs in the BF group at 1 dpf showed evidence of proximal H3K27ac marks. In addition, exposure to a noninfectious bloodmeal resulted in enrichment of immune response genes, likely a preventative measure to protect against potential pathogens present in the bloodmeal (Supplementary Table S3)^63^. Because H2K27ac promotes chromatin accessibility for gene expression, this result supports the hypothesis that bloodfeeding alone sets up favorable regulatory conditions that would favor virus-associated modulation. BF histone modification patterns were dramatically different than SF as early as 1 dpf, eased by 3 dpf and, then by 7 dpf, mosquitoes that had not received a bloodmeal showed substantially higher H3K27ac levels than the other treatment groups, consistent with a possible role in aging. Genes proximal to H3K27ac marks in SF mosquitoes did not change much over the course of time in terms of functional category type (Supplementary Fig. S7).

Overall, these results provide support for the idea that histone modifications are part of the overall response to arbovirus infection in mosquitoes. However, the extent to which specific changes are manipulated by viral proteins or a part of the host defense response must be determined by further experimentation.

## Conclusion

H3K27ac and H3K9me3 marks were altered upon exposure to RVFV MP12, and a subset of histone-proximal DEGs at 3 dpf had associated gene expression changes. Expression of immune genes such as cecropin, transferrin and defensin were significantly upregulated early in infection in a manner consistent with anti-viral defense. Future efforts will continue to explore the epigenetic landscape layers in arbovirus exposed mosquitoes to identify the major players responsible for virus-induced transcriptional repression and whether these changes are consistent across arbovirus systems.

## Materials and methods

### Mosquito and virus experiments

The Poza Rica strain of *Ae. aegypti* was colonized in 2012, originating from the state of Veracruz, Mexico^69^. Mosquito colonies were maintained at 28 °C on a 12:12 light: dark cycle; adults were fed water and sucrose (sugar cubes or raisins) *ad libitum*. Larvae were reared on TetraMin fish food (Spectrum Brands Pet, Blacksburg, VA, USA) ground in a coffee grinder. Mosquitoes (3–7 days old) were starved for food and water for 24 h prior to feeding, then orally exposed to RVFV MP-12 or conditioned cell culture media mixed 1:1 with defibrinated sheep blood for bloodfed controls, as described in Campbell et al.^23^. A high passage strain of MP12 (passage unknown, local lab passage 1), which was a gift from the US Department of Agriculture, was used for these experiments. In brief, freshly grown MP-12 RVFV was grown in Vero cells at a multiplicity of infection of 0.01 for 3 days and then mixed 1:1 in defibrinated calf blood (Colorado Serum Company, Denver, CO, USA), with 1 mM ATP as phagostimulant and orally provided to adult mosquitoes in water-jacketed feeders set to 37 °C. Mosquitoes with visible blood in the abdomen were saved for collection timepoints. Viral bloodmeal titers were at 6.9–7.8 log10 plaque-forming units (PFU) per ml. As a control, a separate group of age-matched mosquitoes were held for the indicated period. Three replicates of each experiment were performed.

Plaque assays were done using methods described previously^23^. Mosquito infection rates were determined as follows. Individual mosquito carcasses, following removal of midguts for CUT&RUN, were placed in 250 µl mosquito diluent (DMEM, 20% heat-inactivated FBS, 50 µg/ml Pen-Strep, 50 µg/ml gentamicin, and 2.5 µg/ml amphotericin B) and stored at -80 °C. Samples were homogenized on a Qiagen Tissuelyzer (Qiagen) at 30 beats per second frequency for 30 s. Homogenates were pelleted for 3 min at 21,000 x g, then 50 µl homogenate was placed into one well of a 12 well plate and assayed in duplicate. At 3 days post-infection, wells were assessed for cytopathic effects. We chose to use carcasses, rather than legs, to measure overall viral load. RVFV has some ‘atypical’ tropisms, as described in early microscopic studies^70^, wherein virus escaped to cardia as early as 1 dpf in susceptible mosquitoes (*Culex*), consistent with bypass of the hemocoel. Also, early experiments of *Aedes aegypti* showed evidence of a dissemination barrier^71^. To validate overall infection status, whole mosquitoes at 7 dpf were homogenized, and traditional plaque assays were performed.

### CUT&RUN

Our approach followed that of Skene, et al.^28^ with modifications. Specifically, pools of 20 midguts from blood-fed and RVFV MP12-fed *Aedes aegypti* were dissected 1, 3 and 7 days post feed or from sugar-fed controls. Midguts were placed into 200 µL of 1x Schneider’s Drosophila Media (Gibco #21720024). Following completion of dissections for all samples, 300 µL of formaldehyde fixation solution was added to each tube for a final concentration of 0.1% (using 16% methanol-free formaldehyde (Cell Signaling # 12606) in phosphate buffered saline (PBS) with 1x protease inhibitor cocktail (PIC, cOmplete™ Protease Inhibitor Cocktail, # 11697498001). After a 10-minute incubation at room temperature, 50 µL of 10x Glycine (Cell Signaling #7005) was added to stop the reactions and samples were incubated for another 5 min at room temperature (RT). Samples were centrifuged for 5 min at 2000xg 4 °C then washed with 500 µL PIC/PBS. At all steps, tubes were flicked to mix solutions rather than pipetting to prevent loss of midguts. Midguts were resuspended in 1 ml Wash buffer (200mM HEPES pH 7.5, 1.5 M NaCl, 0.5 mM spermidine, 1x PIC), then centrifuged for 3 min at 2,000 xg at RT. Each reaction was resuspended in 100 µL 1x Wash buffer. Ten µL per sample of concanavalin A beads (Fisher # NC1831103) were activated using the methods of Skene, et al.^28^. Then the beads were added to the sample and incubated 5 min at RT. Each sample tube was placed on a magnetic stand (ThermoFisher 12321D). Once the solution cleared, the liquid was removed and replaced with 100 µL Digitonin solution (200mM HEPES pH 7.5, 1.5 M NaCl, 20mM spermidine, 1x PIC, 0.1% digitonin) with 2mM EDTA and the appropriate antibody. The antibodies used in this study were 2 µg per reaction anti-H3K27ac Ab (Abcam, ab4729), or 1 µg per reaction anti-H3K9me3 Ab (Abcam, ab8898) and Rabbit (DA1E) mAb IgG XP^®^ Isotype Control (Cell Signaling #66362) as a negative control. Samples were incubated overnight at 4 °C on a rotator. Then the samples were put on the magnetic stand. Once the solution was clear, the samples were washed with 1 ml Digitonin buffer and put back on the stand. Upon removing the buffer, 50 µL solution containing 1.5 µL protein A/G- micrococcal nuclease (pAG/MNase, Epicypher, #15-1116) in Digitonin buffer was added per sample and mixed gently by flicking the tube. Samples were incubated at 4 °C for 1 h. The samples were then washed twice as before in 1 ml Digitonin buffer per sample. Samples were resuspended in 150 µL Digitonin buffer, placed on ice for 5 min, then activated by adding 3 µL 100 mM cold Calcium Chloride to each tube. Samples were incubated at 4 °C for 30 min. Reactions were stopped by the addition of 150 µL Stop buffer (0.17 M NaCl, 10mM EDTA, 2mM EGTA, 0.1 mg/ml glycogen, 0.1% Digitonin, 8.3 mg/ml RNAse A). Samples were the centrifuged at 3 min at 16,000 xg for 2 min. Supernatants were transferred to new tubes containing 3 µL 10% sodium dodecyl sulfate and 2 µl Proteinase K (20 mg/ml). Samples were then incubated at 65 °C for 2 h, then transferred to low bind tubes. DNA fragments were purified using Qiagen PCR purification kits.

### Input sample preparation

Input samples were processed in parallel with the other samples through the first wash steps. Total DNA was extracted using a salt extraction method and resuspended in 100 µl water^72^. Following extraction, samples were placed in a 0.65 mL Diagenode tube and sonicated in a Diagenode Bioruptor Pico (Diagenode) for 20 cycles of 30 s on, 30 s off. Fragment sizes of ~ 200 bp were subsequently validated using an HS D1000 chip with an Agilent 4150 tapestation (Agilent).

### Sequencing

Three biological replicates were sequenced per sample group. Briefly, standard library preparation methods made use of the NEBNext^®^ Ultra™ II DNA Library Prep Kit for Illumina (E7103L) following manufacturer’s directions with 1 ng DNA per library prep and ~ 13 PCR cycles for the amplification step. Library quality was confirmed using high sensitivity DNA reagents (Q33231) on the Qubit 2 (Qubit) and Agilent tapestation 4150 (Agilent). Sequencing was performed on an Illumina NovaSeq (Azenta.com) with read depths indicated in Supplementary Table S2. Libraries with less than 55 nt inserts were removed from analysis (Supplementary Table S2).

### Analysis

All scripts utilized for the analysis of this data can be found on GitHub (https://github.com/CRosenbergCode/Aedes_aegypti_Midgut_CUT-RUN), Sequencing reads were trimmed and filtered using Fastp version 0.23.4^73^. Duplicate reads were removed for ChIP-seq reads but retained for RNA-seq analysis^74^. These trimmed and filtered reads were aligned to an index prepared from the VectorBase-68_AaegyptiLVP_AGWG_Genome.fasta using HISAT2 version 2.2.1^75,76^. Only paired reads which aligned concordantly were retained. Data regarding alignment rates and quality parameters per sample can be found in Supplementary Tables S1-3. Peak calling was performed on merged alignments using MACS2 macs2 2.2.9.1 using --keep dups all, qvalue 0.05 against VectorBase-68_AaegyptiLVP_AGWG_Genome.fasta^76,77^. All samples were standardized using the corresponding input sample.

Bigwig files were created using Deeptools bamCoverage with a bin size of 10 bases and normalized using reads per genome coverage (RPGC)^78^. They were then compared against their corresponding inputs using Deeptools bigwigCompare, with a bin size of 10 bases and --operation subtract, thus generating bigwigs that use direct subtraction as opposed to fold change. Peak heatmaps and read density maps were made using deepTools functions “computeMatrix”, and “plotHeatmap”. computeMatrix was run with the “make_matrices.sh” script using VectorBase-68_AaegyptiLVP_AGWG_Genome.bed (No MIT, long-noncoding RNAs or pseudogenes) as reference.

Differential binding analysis was performed using Diffbind version 3.12.0 with the edgeR option^79^ in R version 4.3.2^80^ using a false discovery rate(FDR) threshold of *p* < 0.10. A custom script was created to determine the proximity of DiffBind peaks to genes or other features of interest in the *Ae. aegypti* genome (Supplementary Tables S4 and S5). Venn diagram were produced using Venny 2.1^81^.

### RNA sequencing and analysis

Three to 4 biological replicates were sequenced per sample group. Bulk RNA-Seq libraries were prepared from 200 ng total RNA, using polyA+ purification (NEBNext^®^ Poly(A) mRNA Magnetic Isolation Module) prior to library prep with the NEBNext^®^ Ultra™ II Directional RNA Library Prep Kit for Illumina. Sequencing was performed on an Illumina NovaSeq (Azenta.com) with read depths indicated in Supplementary Table S1.

For RNA-seq alignment, a reference transcriptome was created using VectorBase-68_AaegyptiLVP_AGWG_AnnotatedTranscripts.fasta^76^ using HISAT2 (2.2.1)^75^, then alignment was performed using standard parameters. Gene-level aggregation was performed using HTseq-count (2.0.5). Differential expression analysis was performed using DEseq2 (1.48.0).

### Gene set enrichment analysis

GO-GSEA libraries were built using AnnotationForge version 2.13 in R. Genome-wide annotation was performed using Entrez gene identifiers^38^. The fast gene set enrichment analysis algorithm R implemented by the fgsea R package (version 1.28.0)^82^ was used for GO-GSEA analysis^83^. GO terms were obtained using EggNog Mapper 2.1.12, limiting the orthology search to Diptera only^36^. Custom annotations were utilized to define broader functional categories as described in Campbell et al. 2019^32^. For both GSEA (ranked nonparametric analysis) and ORA (simple arithmetical calculation that does not involve ranking), a minimum size of 15 and a maximum size of 500 were required for a GO annotation to be included in analysis. Ranking was based on the -log10(p-value). In all cases, ordering was decided arbitrarily in case of ties. GO-GSEA of RNA-seq differential binding was based on DESeq2 results. GO-GSEA of ChIP-seq differential binding was based on Diffbind results. In cases of multiple peaks corresponding to the same gene, only the peak with the highest p-value was retained. For ORA, duplicate peaks for the same gene were removed to ensure each gene only occurred once. For analysis of promoters, only peaks within 2000 bp of the TSS were included for the analysis. For analysis of enhancers, only peaks between 50,000 and 200,000 bp away from a TSS that were annotated as distal using ChIPSeeker were included for the analysis^84,85^. The custom annotation was manually compiled based on Campbell et al. 2019, which relied on VectorBase orthological assignments^32,76^.The custom OrgDB database and GMT files used are provided on the github for this manuscript. Visualization of gene enrichment utilized enrichplot version 1.28^86^ and ClusterProfiler version 4.15^87^.

### Motif enrichment analysis

Enrichment analysis was performed using HOMER (Hypergeometric Optimization of Motif EnRichment) version 5.1 with a custom genome created using VectorBase-68_AaegyptiLVP_AGWG annotations^88,89^. An offset of 2000 base pairs upstream and 200 base pairs downstream of the transcription start site were used and a random selection of promoter regions was used as the background. For searching known motifs such as CI/GLI binding sites, the consensus sequence (GACCACCCA) in *D. melanogaster* was used and a maximum of 1 mismatch was allowed. Genes from this study with these motifs are in Supplementary Table S8.

### PCR validation

Select DEGs shown in Supplementary Table S7 were subjected to reverse transcriptase quantitative PCR using methods described elsewhere^4^. Samples from 7 dpf were collected from RVFV-exposed, BF or SF groups. Primers were listed in Supplementary Table S9. Relative quantitation using BF samples as calibrators was executed using established methods^90^.

## Supplementary Information

Below is the link to the electronic supplementary material.

Supplementary Material 1

## Data Availability

All raw sequencing data has been uploaded to the NCBI Sequence Read archive under Bioprojects PRJNA1284329 and PRJNA1290393.
