# Supplementary material for: Altered histone modifications in Aedes aegypti midguts following Rift Valley fever virus exposure
Source: Sci Rep. 2026 Jan 29;16:6605. doi: 10.1038/s41598-026-37729-y (PMC12913953; doi:10.1038/s41598-026-37729-y)
Supplement: Supplementary file 1 — Supplementary Material 1 [file 41598_2026_37729_MOESM1_ESM.zip › CutRun_ms_Suppl_Figs_revision.pptx]

## Slide 1
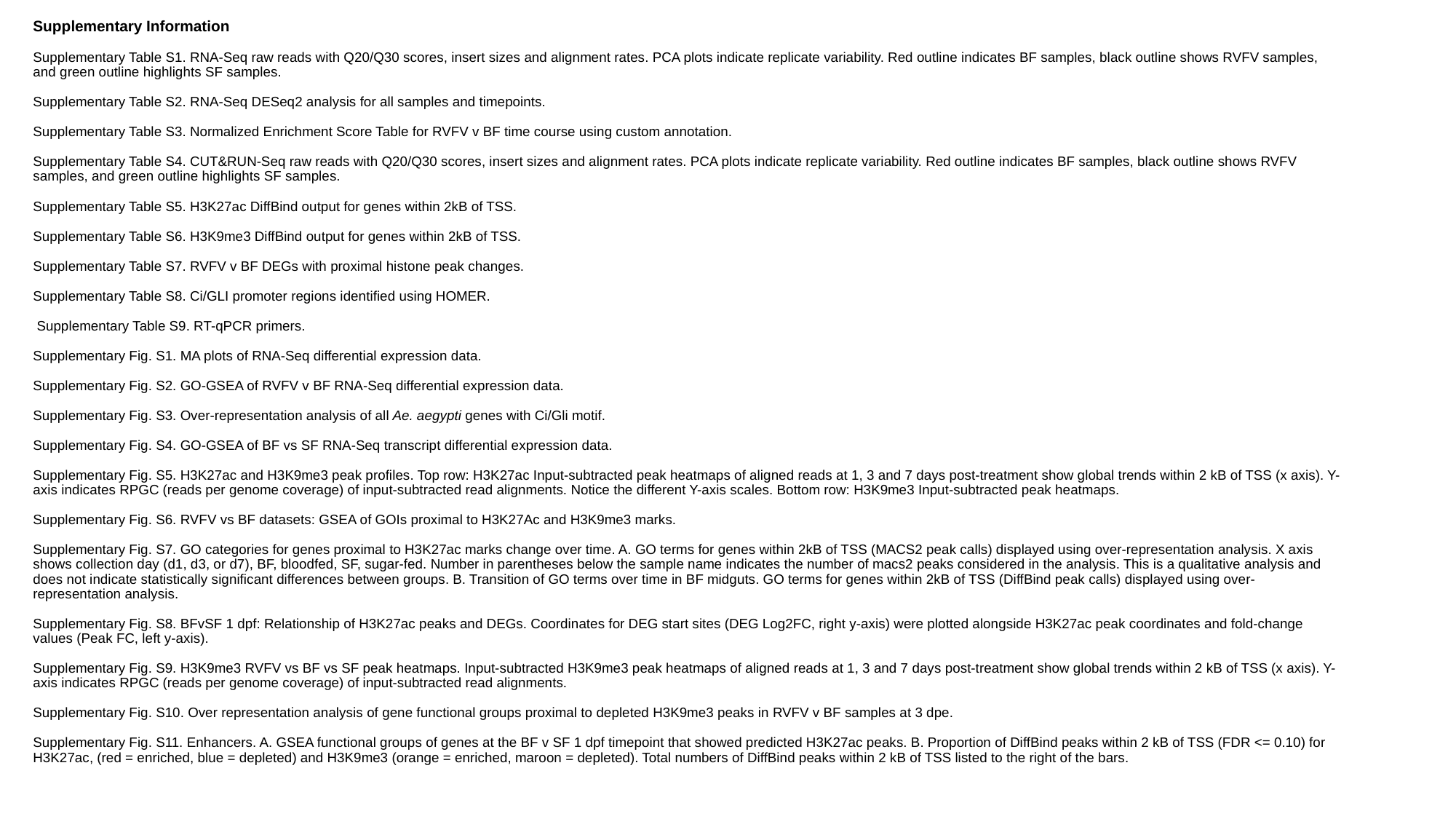

Supplementary Information
Supplementary Table S1. RNA-Seq raw reads with Q20/Q30 scores, insert sizes and alignment rates. PCA plots indicate replicate variability. Red outline indicates BF samples, black outline shows RVFV samples, and green outline highlights SF samples.
Supplementary Table S2. RNA-Seq DESeq2 analysis for all samples and timepoints.
Supplementary Table S3. Normalized Enrichment Score Table for RVFV v BF time course using custom annotation.
Supplementary Table S4. CUT&RUN-Seq raw reads with Q20/Q30 scores, insert sizes and alignment rates. PCA plots indicate replicate variability. Red outline indicates BF samples, black outline shows RVFV samples, and green outline highlights SF samples.
Supplementary Table S5. H3K27ac DiffBind output for genes within 2kB of TSS.
Supplementary Table S6. H3K9me3 DiffBind output for genes within 2kB of TSS.
Supplementary Table S7. RVFV v BF DEGs with proximal histone peak changes.
Supplementary Table S8. Ci/GLI promoter regions identified using HOMER.
 Supplementary Table S9. RT-qPCR primers.
Supplementary Fig. S1. MA plots of RNA-Seq differential expression data.
Supplementary Fig. S2. GO-GSEA of RVFV v BF RNA-Seq differential expression data.
Supplementary Fig. S3. Over-representation analysis of all Ae. aegypti genes with Ci/Gli motif.
Supplementary Fig. S4. GO-GSEA of BF vs SF RNA-Seq transcript differential expression data.
Supplementary Fig. S5. H3K27ac and H3K9me3 peak profiles. Top row: H3K27ac Input-subtracted peak heatmaps of aligned reads at 1, 3 and 7 days post-treatment show global trends within 2 kB of TSS (x axis). Y-axis indicates RPGC (reads per genome coverage) of input-subtracted read alignments. Notice the different Y-axis scales. Bottom row: H3K9me3 Input-subtracted peak heatmaps.
Supplementary Fig. S6. RVFV vs BF datasets: GSEA of GOIs proximal to H3K27Ac and H3K9me3 marks.
Supplementary Fig. S7. GO categories for genes proximal to H3K27ac marks change over time. A. GO terms for genes within 2kB of TSS (MACS2 peak calls) displayed using over-representation analysis. X axis shows collection day (d1, d3, or d7), BF, bloodfed, SF, sugar-fed. Number in parentheses below the sample name indicates the number of macs2 peaks considered in the analysis. This is a qualitative analysis and does not indicate statistically significant differences between groups. B. Transition of GO terms over time in BF midguts. GO terms for genes within 2kB of TSS (DiffBind peak calls) displayed using over-representation analysis.
Supplementary Fig. S8. BFvSF 1 dpf: Relationship of H3K27ac peaks and DEGs. Coordinates for DEG start sites (DEG Log2FC, right y-axis) were plotted alongside H3K27ac peak coordinates and fold-change values (Peak FC, left y-axis).
Supplementary Fig. S9. H3K9me3 RVFV vs BF vs SF peak heatmaps. Input-subtracted H3K9me3 peak heatmaps of aligned reads at 1, 3 and 7 days post-treatment show global trends within 2 kB of TSS (x axis). Y-axis indicates RPGC (reads per genome coverage) of input-subtracted read alignments.
Supplementary Fig. S10. Over representation analysis of gene functional groups proximal to depleted H3K9me3 peaks in RVFV v BF samples at 3 dpe.
Supplementary Fig. S11. Enhancers. A. GSEA functional groups of genes at the BF v SF 1 dpf timepoint that showed predicted H3K27ac peaks. B. Proportion of DiffBind peaks within 2 kB of TSS (FDR <= 0.10) for H3K27ac, (red = enriched, blue = depleted) and H3K9me3 (orange = enriched, maroon = depleted). Total numbers of DiffBind peaks within 2 kB of TSS listed to the right of the bars.

## Slide 2
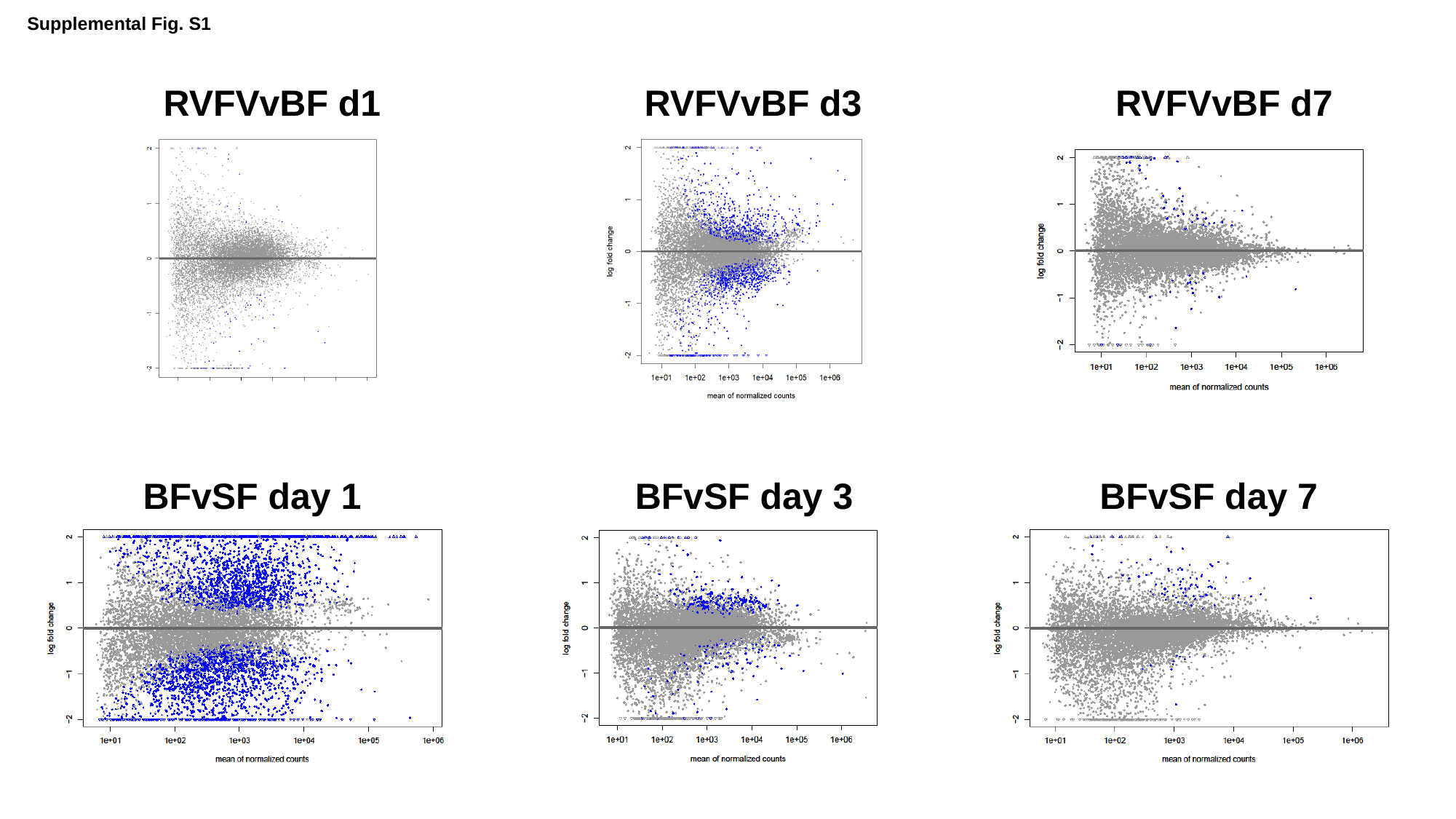

Supplemental Fig. S1
 RVFVvBF d1 RVFVvBF d3 RVFVvBF d7
 BFvSF day 1 BFvSF day 3	 BFvSF day 7

## Slide 3
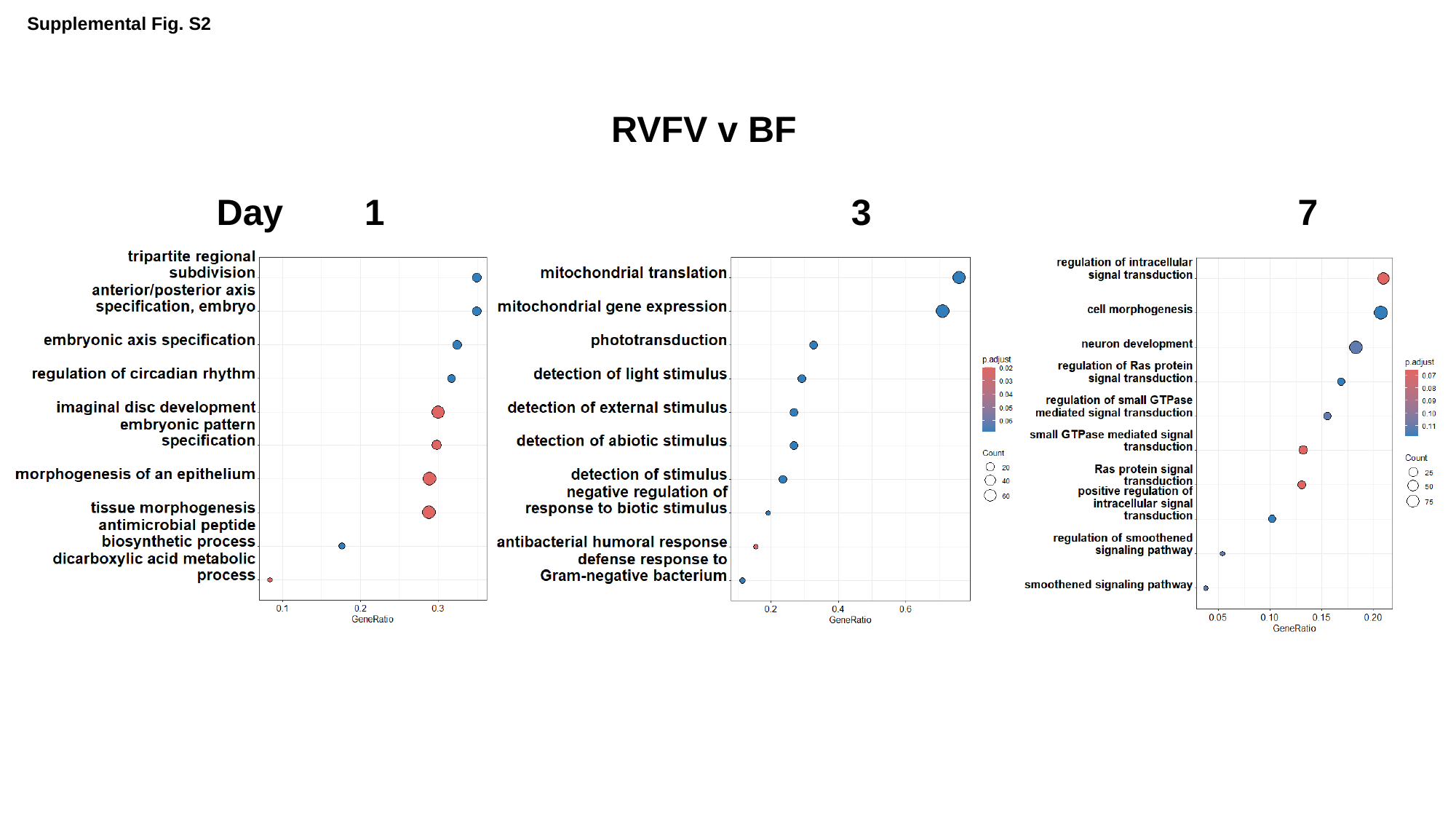

Supplemental Fig. S2
RVFV v BF
Day 1 3 7

## Slide 4
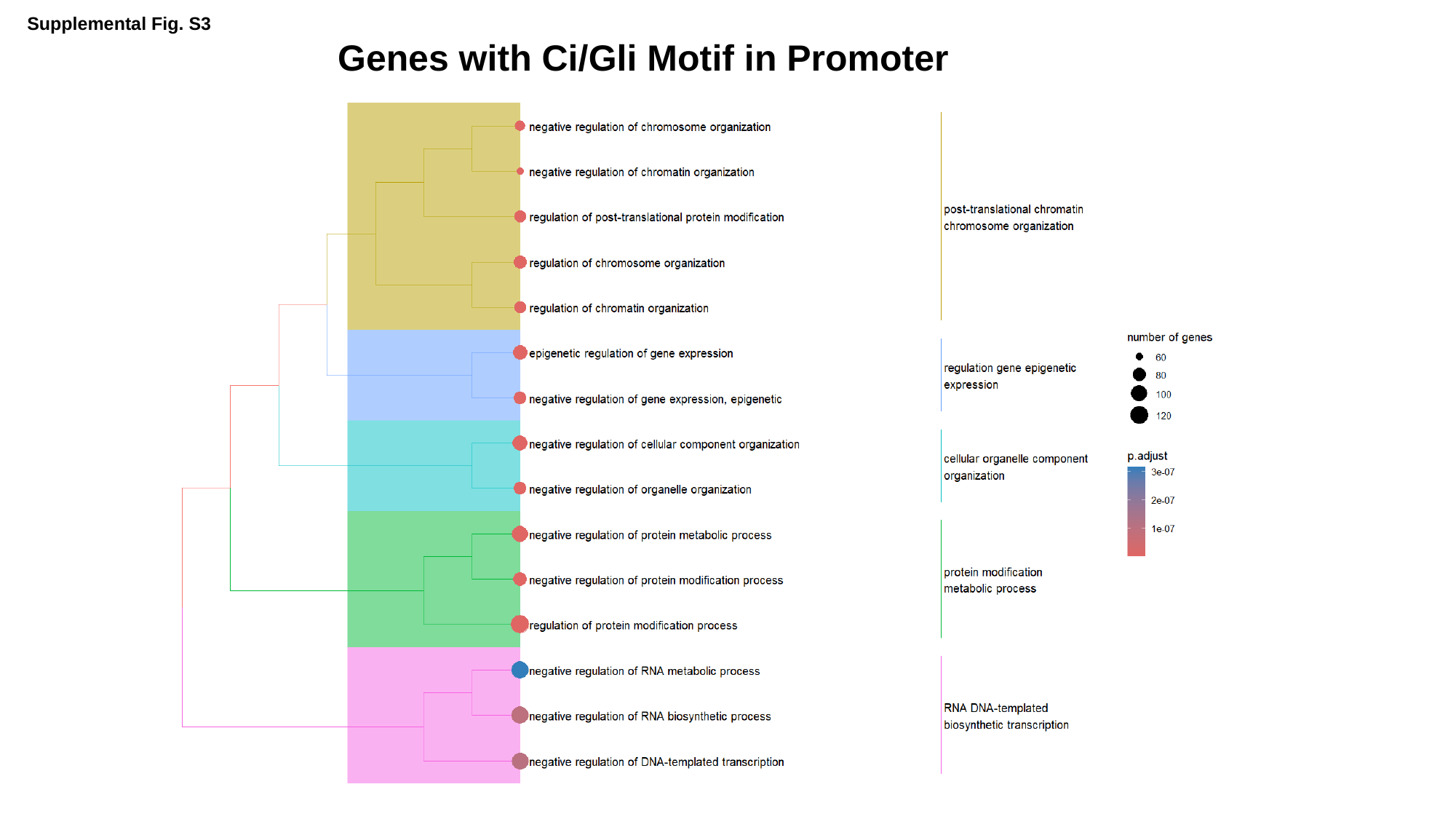

Supplemental Fig. S3
Genes with Ci/Gli Motif in Promoter

## Slide 5
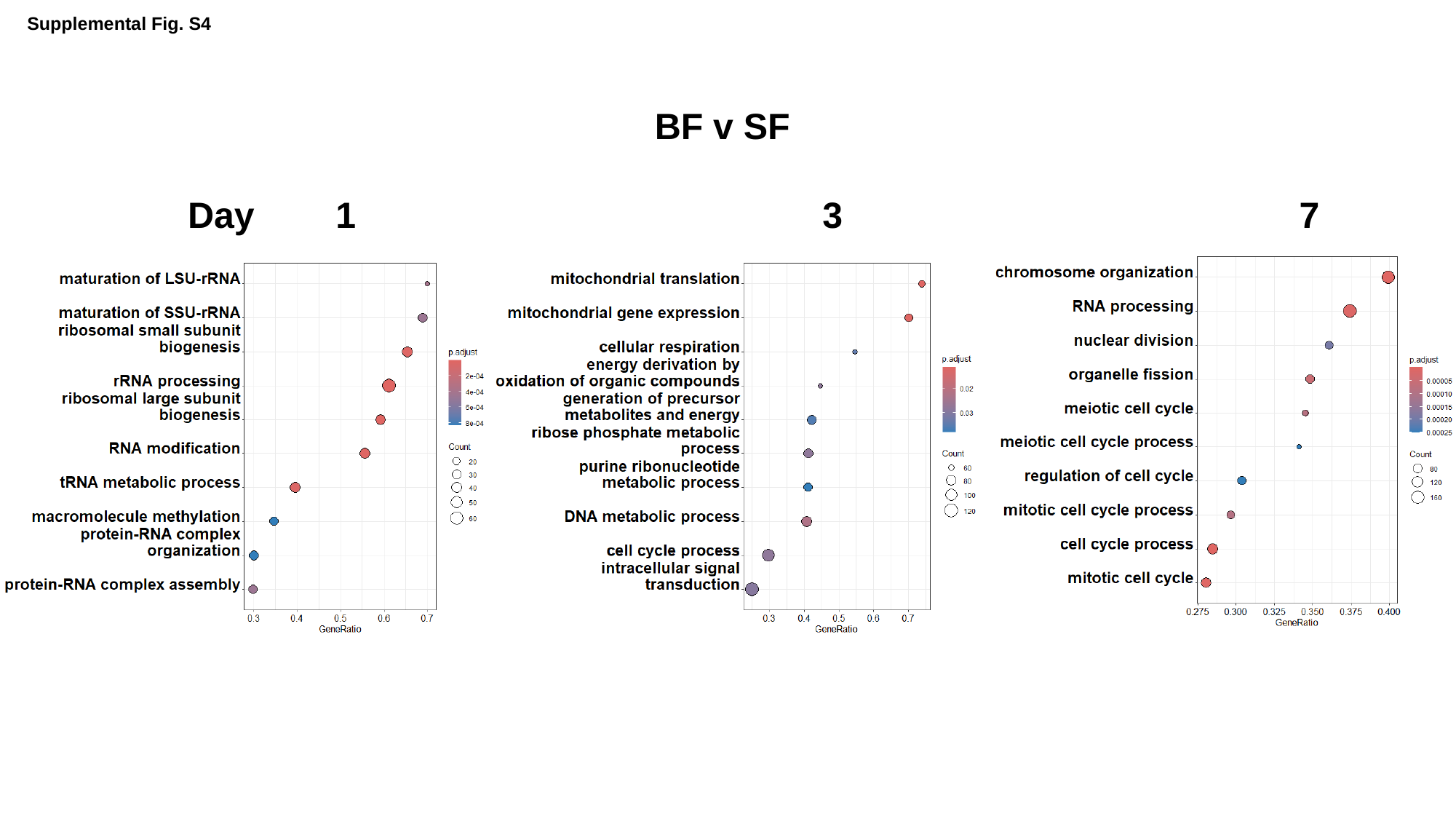

Supplemental Fig. S4
BF v SF
Day 1 3 7

## Slide 6
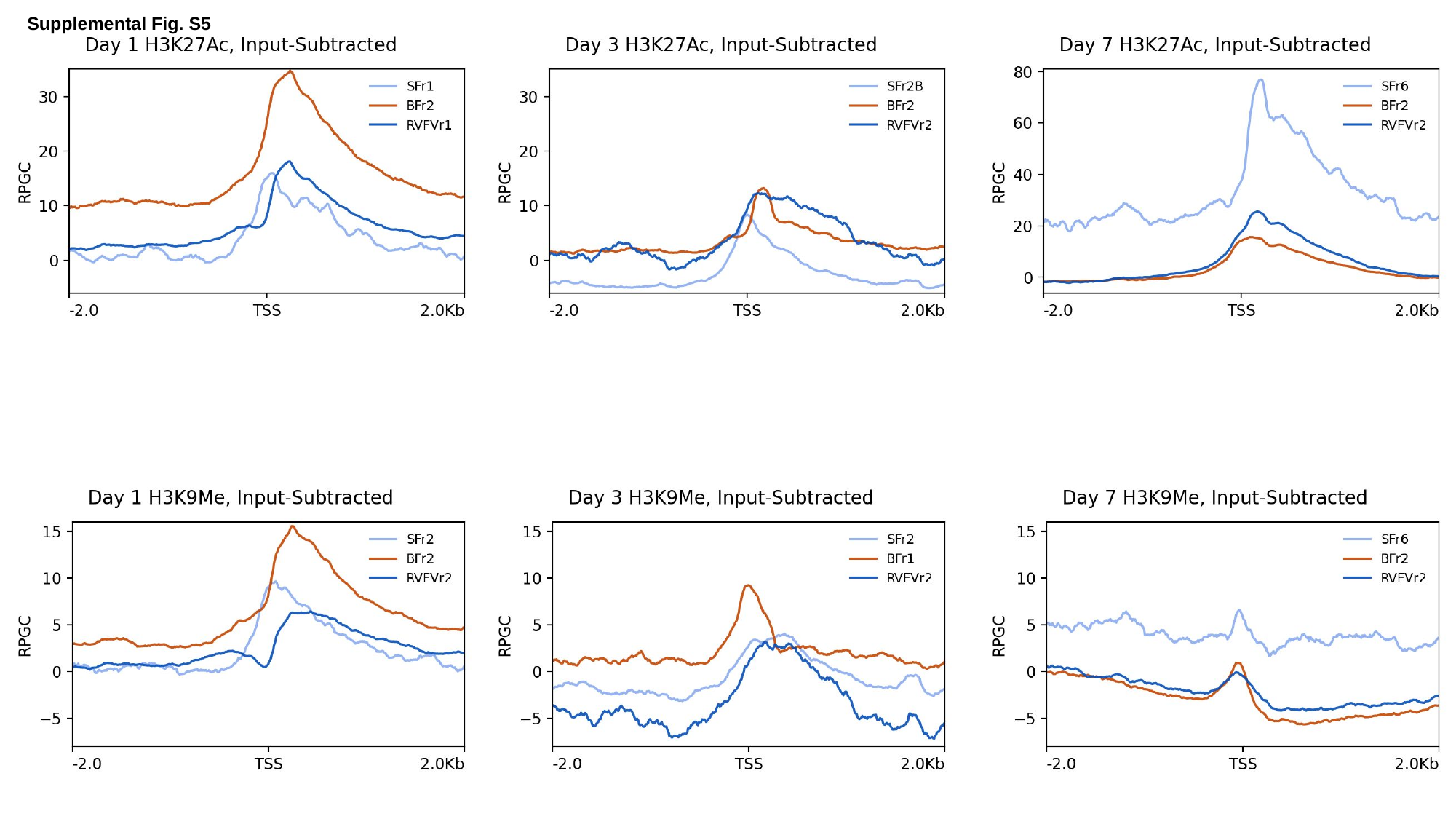

Supplemental Fig. S5

## Slide 7
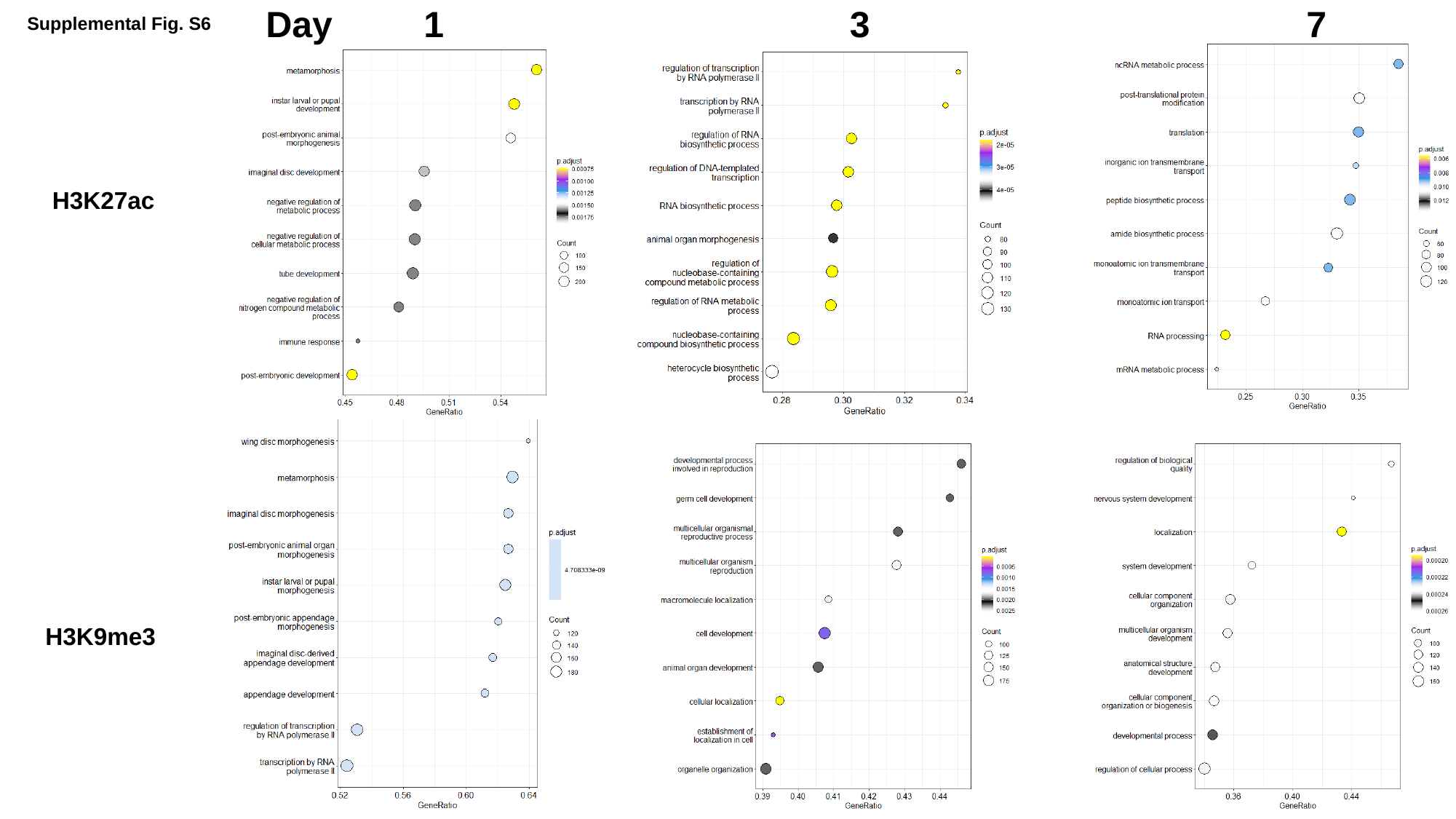

Day 1 3 7
Supplemental Fig. S6
 H3K27ac
 H3K9me3

## Slide 8
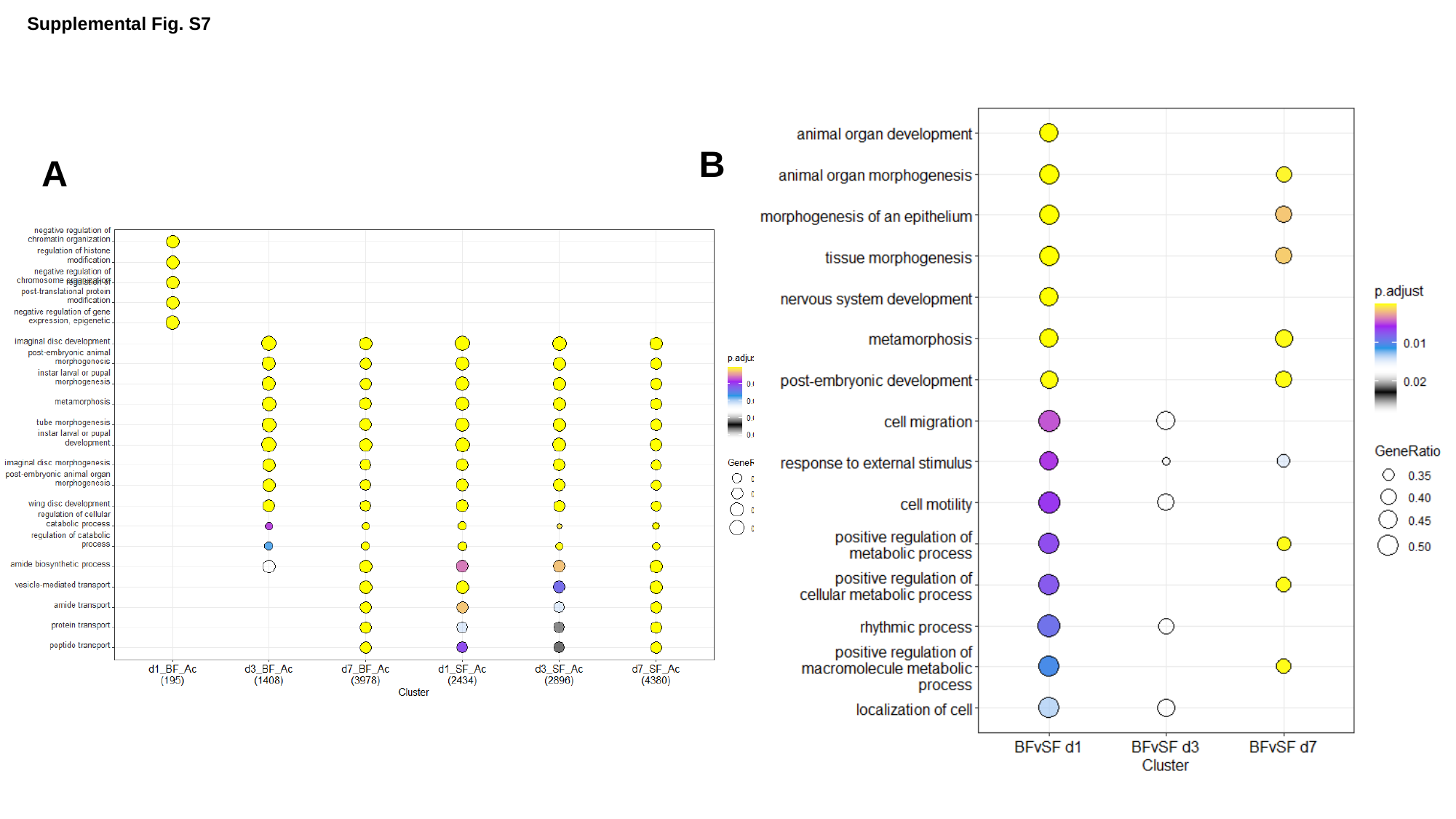

Supplemental Fig. S7
B
A

## Slide 9
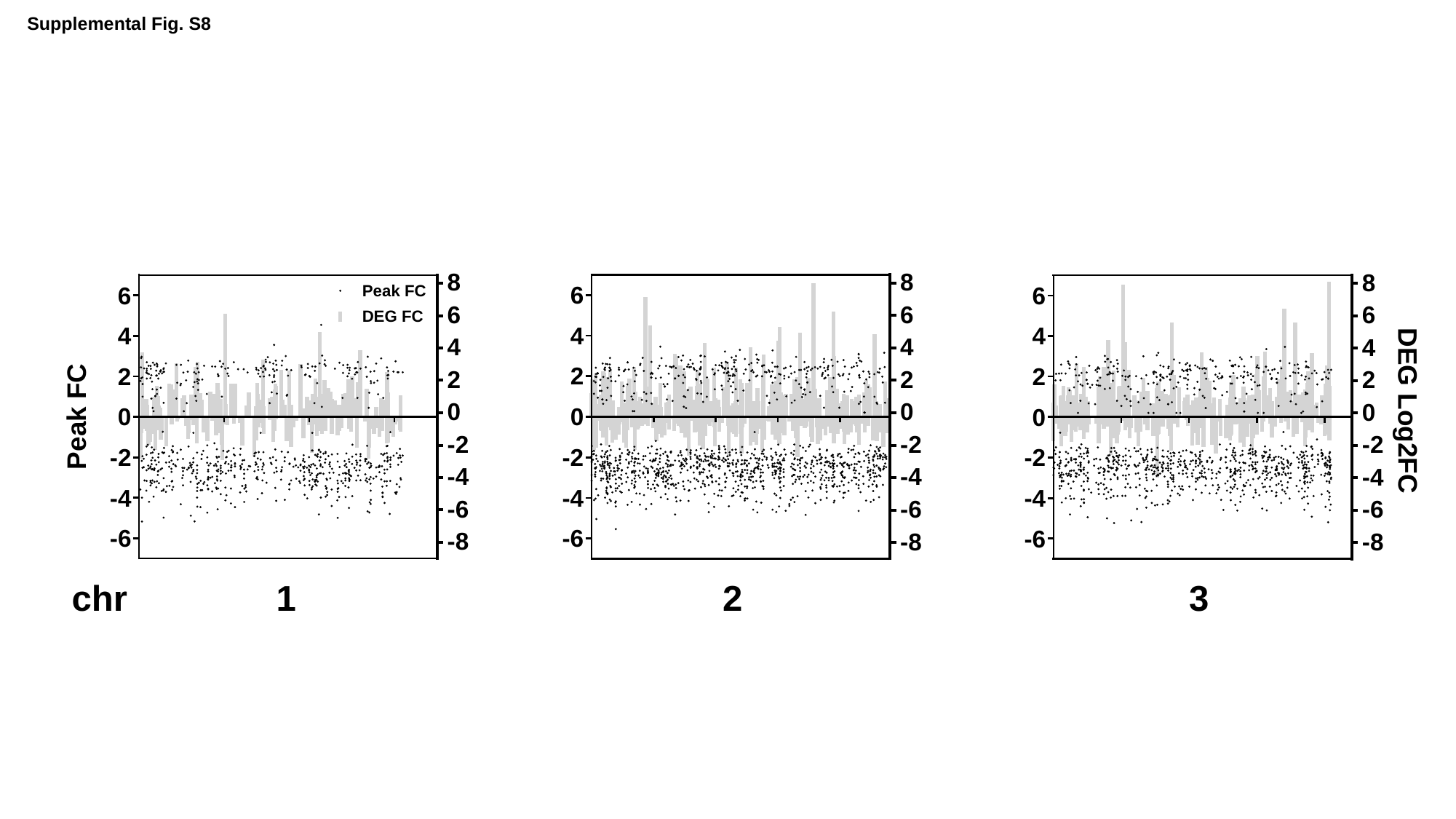

Supplemental Fig. S8

## Slide 10
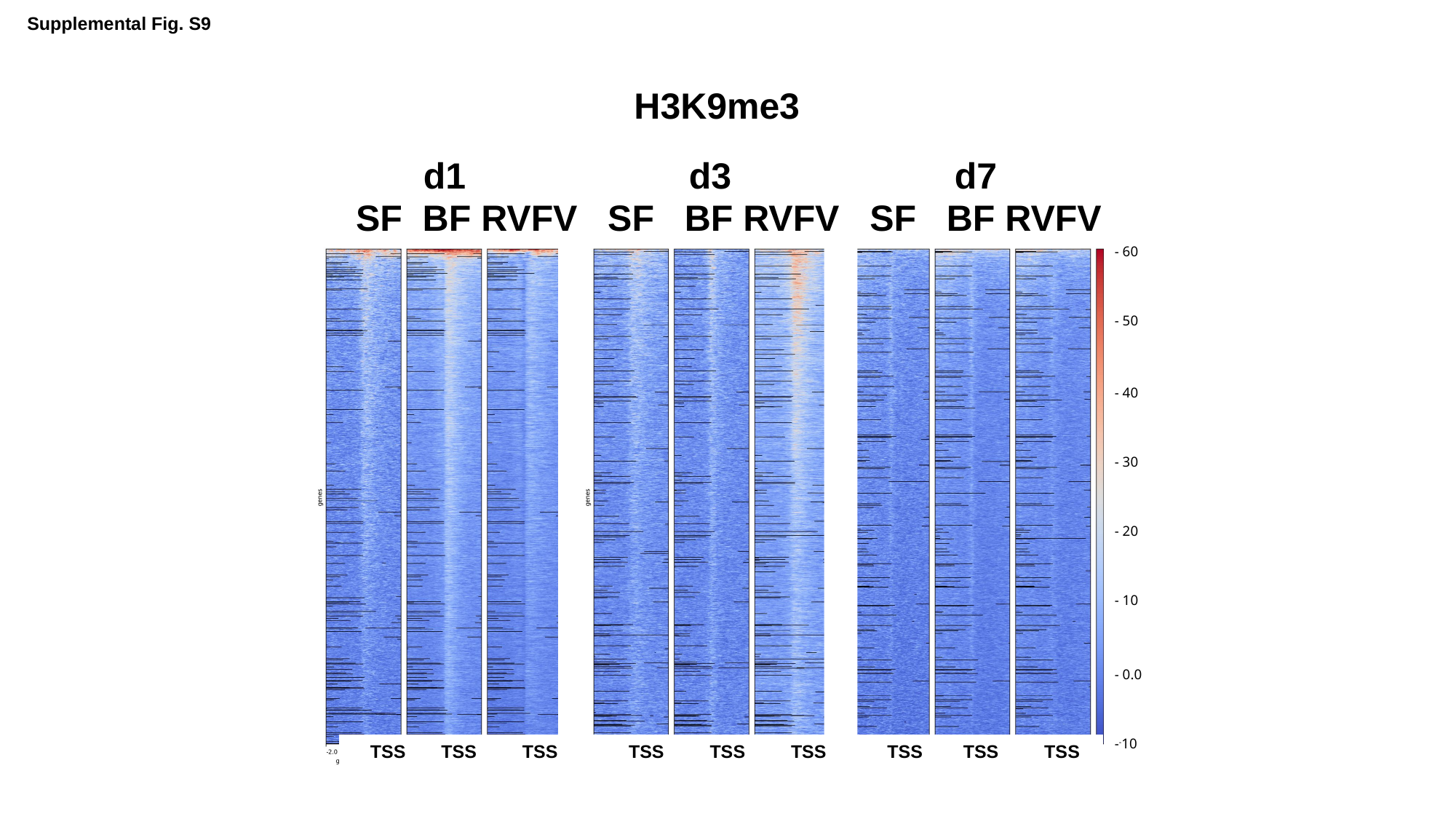

Supplemental Fig. S9
H3K9me3
H3K9me3
# d1 d3 d7
SF BF RVFV SF BF RVFV SF BF RVFV
- 60
- 50
- 40
- 30
- 20
- 10
- 0.0
--10
 TSS TSS TSS TSS TSS TSS TSS TSS TSS

## Slide 11
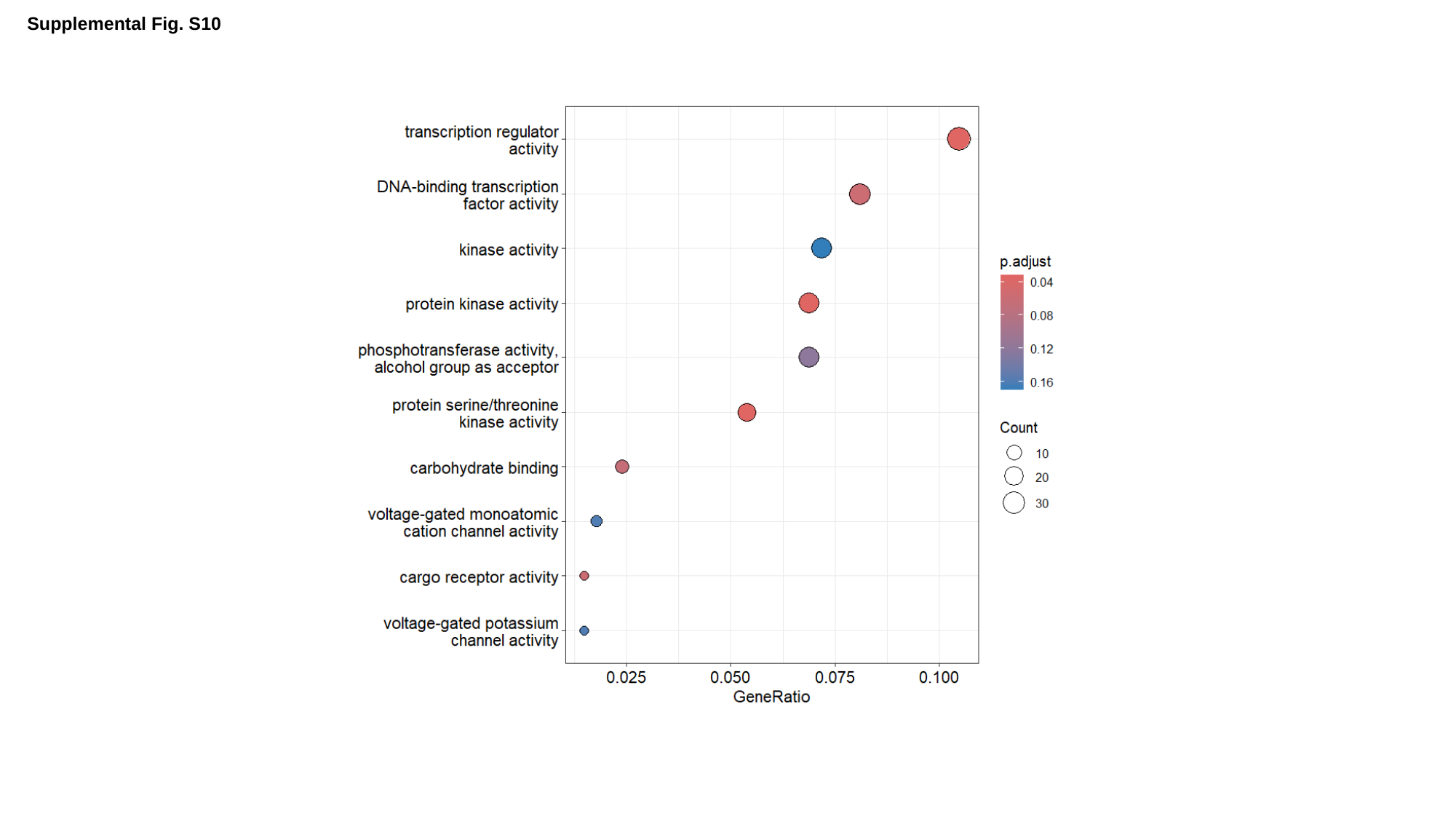

Supplemental Fig. S10

## Slide 12
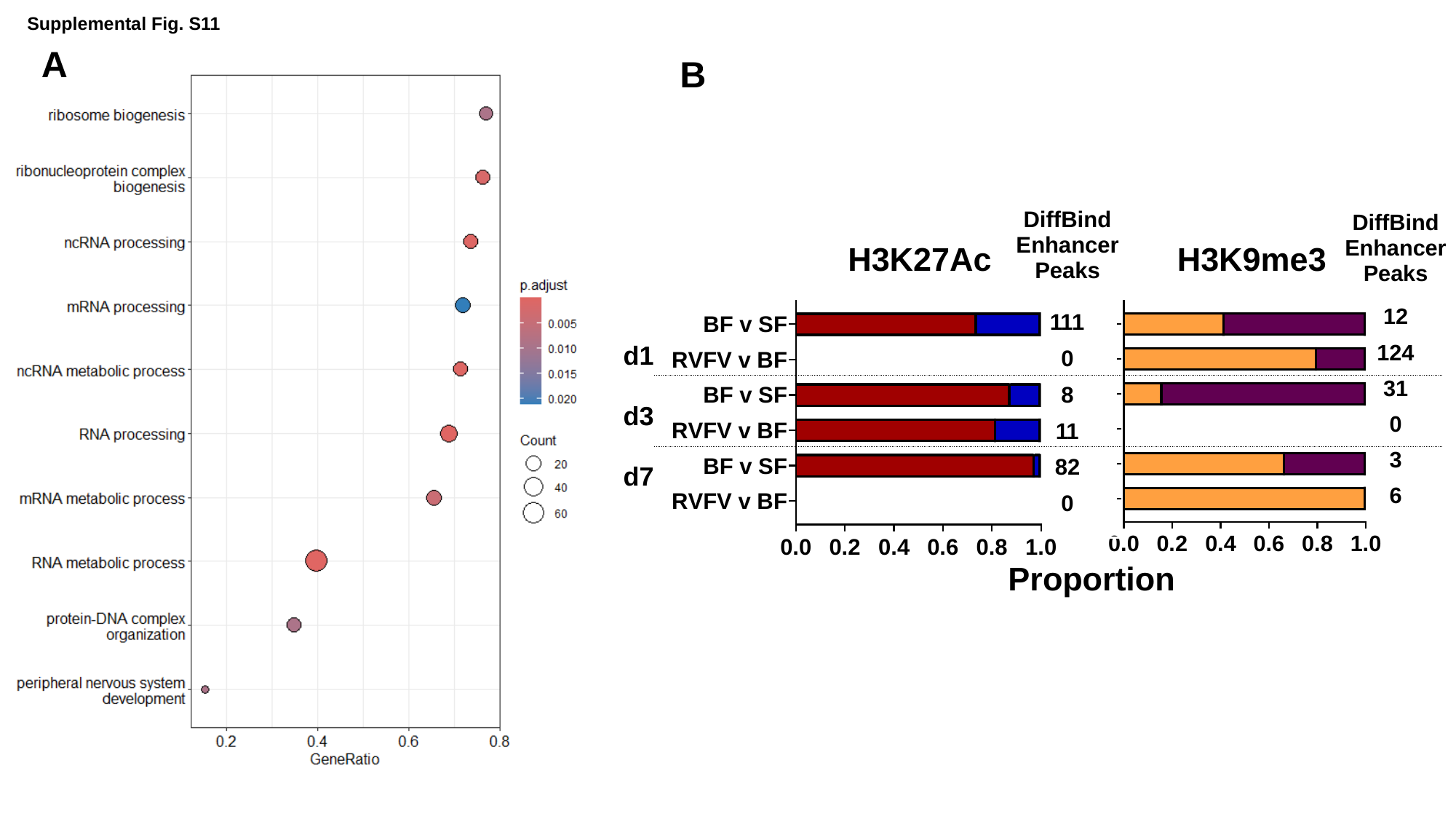

Supplemental Fig. S11
A
B
